# Impact on Endovascular Thrombectomy for Acute Ischemic Stroke of Aortic Arch Calcification on Chest X-ray

**DOI:** 10.3390/jcm12196115

**Published:** 2023-09-22

**Authors:** Hyeon Yeong Jeong, Taek Min Nam, Sang Hyuk Lee, Ji Hwan Jang, Young Zoon Kim, Kyu Hong Kim, Kyeong Hwa Ryu, Do-Hyung Kim, Byung Soo Kwan, Hyerang Bak, Seung Hwan Kim

**Affiliations:** 1 Department of Neurosurgery, Samsung Changwon Hospital, Sungkyunkwan University School of Medicine, Changwon 51353, Republic of Korea; wjdgusdud23@naver.com (H.Y.J.); taekmin82@gmail.com (T.M.N.); shlee858@naver.com (S.H.L.); gebassist@naver.com (J.H.J.); yzkim@skku.edu (Y.Z.K.); ppopei@naver.com (K.H.K.); 2 Department of Radiology, Samsung Changwon Hospital, Sungkyunkwan University School of Medicine, Changwon 51353, Republic of Korea; ryukh0329@gmail.com; 3 Department of Neurology, Samsung Changwon Hospital, Sungkyunkwan University School of Medicine, Changwon 51353, Republic of Korea; pons1217@gmail.com; 4 Department of Internal Medicine, Samsung Changwon Hospital, Sungkyunkwan University School of Medicine, Changwon 51353, Republic of Korea; kbs2459@naver.com; 5 Department of Family Medicine, Samsung Changwon Hospital, Sungkyunkwan University School of Medicine, Changwon 51353, Republic of Korea; hyerangbak@gmail.com

**Keywords:** aortic arch, calcification, endovascular thrombectomy, acute ischemic stroke, successful recanalization

## Abstract

Background: Vascular conditions can affect the recanalization rates after endovascular thrombectomy (EVT) for acute ischemic stroke (AIS). Chest radiography can assess the conditions of the aortic arch based on the presence or absence of aortic arch calcification (AoAC). The aim of this study was to investigate the relationship between AoAC on chest radiography and first-pass successful recanalization (modified thrombolysis in cerebral infarction 2b/3 after the first-pass). Methods: We compared the rate of first-pass successful recanalization between patients with and without AoAC. A total of 193 patients with anterior circulation occlusion who underwent EVT between January 2017 and December 2021 were included. Results: AoAC was observed in 80 (41.5%) patients. Patients with AoAC were older (74.5 ± 7.78 vs. 63.9 ± 12.4 years, *p* < 0.001), had more EVT attempts (3.04 ± 1.95 vs. 2.01 ± 1.34 times, *p* < 0.001), and a longer procedural time (71.7 ± 31.2 vs. 48.7 ± 23.1 min, *p* < 0.001) than those without AoAC. Moreover, Patients with AoAC showed a lower incidence of first-pass successful recanalization (18.8% vs. 47.8%, *p* < 0.001) and a higher incidence of postprocedural hemorrhage (45.0% vs. 27.7%, *p* = 0.015) than those without AoAC. On multivariate analysis, AoAC was independently associated with first-pass successful recanalization (odds ratio: 0.239 [0.121–0.475], *p* < 0.001). Conclusions: AoAC on chest radiography can be used as a preoperative predictor of successful first-pass recanalization in patients undergoing EVT for AIS.

## 1. Introduction

Several randomized clinical trials have established the clinical efficacy of endovascular thrombectomy (EVT) for acute ischemic stroke (AIS) [1,2,3,4,5]. Successful recanalization is the most critical prognostic factor for good clinical outcomes in patients with AIS [6,7]; therefore, achievement of a modified Thrombolysis in Cerebral Infarction (mTICI) score of 2b or 3 is the goal of EVT for AIS [7,8,9].

Recent retrospective studies have reported a relationship between the number of device passes and clinical outcomes and documented that recanalization with fewer device passes is associated with better clinical outcomes [10,11,12,13,14,15]. Some studies have also shown that recanalization with multiple device passes can improve the rate of successful recanalization but not the rate of good clinical outcomes [10,12,16]. Therefore, first-pass recanalization has recently become the goal of EVT for AIS [15].

Intracranial atherosclerotic stenosis (ICAS) and tortuous vascular anatomy are common causes of EVT failure [17,18,19]. ICAS-related large vessel occlusions are associated with procedural complexity, a higher re-occlusion rate, and a greater need for rescue treatments, resulting in relatively poor clinical and radiological outcomes [17]. Severe vascular tortuosity can cause stent retrievers to stretch or collapse [18] and can also cause aspiration catheters to reduce contact between the aspiration catheter and thrombus, resulting in a lower chance of first-pass recanalization [18].

Atherosclerosis is a diffuse progressive disease characterized by vascular calcification, which is associated with disease progression [20,21,22]. Chest radiography can assess the condition of the aortic arch based on the presence or absence of aortic arch calcification (AoAC) [22]. Given that AoAC on chest radiographs is associated with an unfavorable aortic arch type, it can also provide information regarding tortuous vascular conditions [20,23].

The aim of this study was to investigate the relationship between AoAC on chest radiography and first-pass recanalization after EVT for AIS.

## 2. Materials and Methods

### 2.1. Study Population

**Figure 1:**
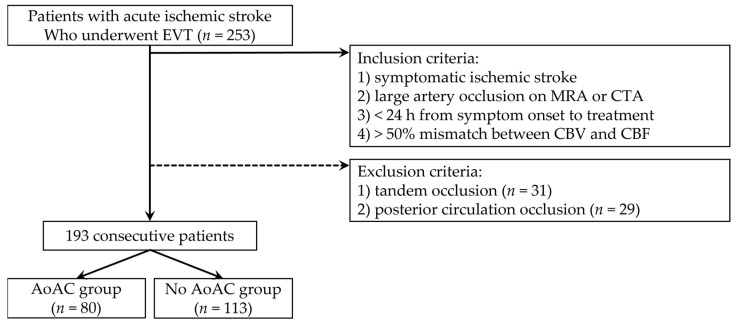
Case accrual process flowchart. (EVT, endovascular thrombectomy; MRA, magnetic resonance angiography; CTA, computed tomography angiography; CBV, cerebral blood volume; CBF, cerebral blood flow; AoAC, aortic arch calcification).

We retrospectively collected data from patients who underwent EVT for AIS at our hospital between January 2017 and December 2021. The inclusion criteria were as follows: (1) symptomatic AIS, (2) large vessel occlusions confirmed using magnetic resonance (MR) angiography or computed tomography (CT) angiography, (3) less than 24 h from symptom onset to treatment, and (4) at least one-half mismatch between cerebral blood flow and cerebral blood volume map with MR perfusion imaging. A total of 253 patients who met the inclusion criteria were included. From the 253 patients, 60 were excluded because of (1) tandem occlusions (*n* = 31) and (2) posterior circulation occlusions (*n* = 29). Eventually, 193 consecutive patients were included (Figure 1).

### 2.2. Data Collection

After obtaining approval from the institutional review board (SCMC 2022-08-003), the imaging data and medical records of patients were reviewed. Data regarding the baseline characteristics of the patients, treatment characteristics, and radiological and clinical outcomes were obtained from the medical records. Baseline characteristics included age, sex, past medical history, occlusion side and site, etiology of stroke, AoAC on chest X-ray, type of aortic arch, National Institutes of Health Stroke Scale (NIHSS) at admission, onset-to-door time, and onset-to-puncture time. Treatment characteristics included intravenous thrombolysis, balloon guide catheter, EVT technique, and procedural time. Radiological and clinical outcomes included TICI grades, postprocedural hemorrhage, and the 3-month modified Rankin Scale (mRS) score.

**Figure 2:**
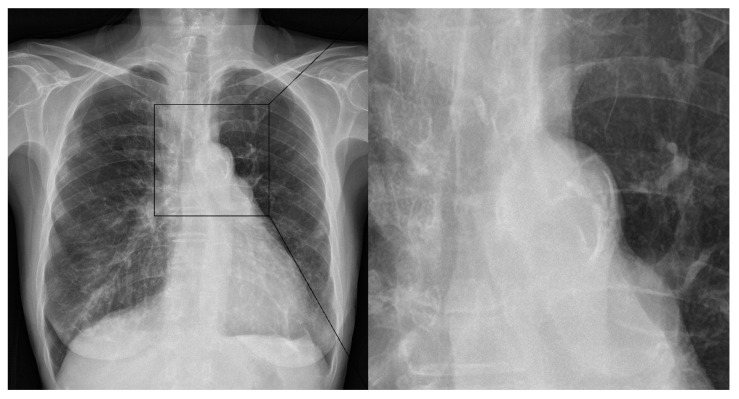
Assessment of aortic arch calcification using chest X-ray. Aortic arch calcification is assessed based on the presence of calcification.

The occlusion sites were divided into three categories: (1) internal carotid artery (ICA), (2) M1 segment of the middle cerebral artery (MCA), and (3) M2 segment of MCA. The M1 and M2 segments were defined as the horizontal and vertical segments of MCA, respectively. The etiology of stroke was classified based on the Trial of ORG 10172 in Acute Stroke Treatment (TOAST) criteria. Two experienced radiologists (7 (KHR) and 6 (JB) years of experience) independently evaluated all chest X-rays in a binary manner. During the analysis, all radiologists were blinded to the radiological and clinical outcomes after EVT. AoAC assessments on chest radiographs are shown in Figure 2. Type of aortic arch was classified according to the vertical distance from the brachiocephalic artery to the apex of the arch: Type 1, a distance of <1 diameter of the left common carotid artery (CCA); Type 2, the distance between 1 and 2 CCA diameters; and Type 3, a distance of >2 CCA diameters [23]. ICAS was diagnosed if there was stenosis of >50% or occlusion without the presence of cardioembolic sources [24]. Patients admitted within 4.5 h of the onset of AIS symptoms were suitable candidates for intravenous administration of tissue plasminogen activator. EVT was performed using a stent retriever, catheter aspiration, or a combination of both. The EVT technique was divided into combination and non-combination techniques. The procedural time was defined as the total time from puncture to recanalization. Successful recanalization was defined by an mTICI grade of 2b or 3. First-pass successful recanalization was defined as an mTICI grade of 2b or 3 after the first-pass. Postprocedural hemorrhage included subarachnoid hemorrhage (SAH) and intracerebral hematoma (ICH), according to the European Cooperative Acute Stroke Study classification. A good clinical outcome was defined by a 3-month mRS score of 0–2.

### 2.3. EVT for AIS

There were three neurosurgeons who specialized in neurointerventions at our hospital. When EVT was planned for large vessel occlusion, it was performed by one of these three neurosurgeons. The procedures were performed under local anesthesia via femoral access. The primary EVT modality was at the surgeon’s discretion. The use of a balloon guide catheter (Flowgate; Stryker Neurovascular, Fremont, CA, USA) was based on the surgeon’s discretion.

### 2.4. Statistical Analysis

Baseline characteristics, treatment characteristics, and radiological and clinical outcomes were compared between patients with AoAC and those without, between patients first-pass successful recanalization and those without, and between patients with good clinical outcomes and those without. Categorical variables were analyzed using the chi-squared test or Fisher’s exact test. Continuous variables were analyzed using Student’s t-test or the Mann–Whitney U test. A *p*-value of <0.05 was considered statistically significant. Multivariable logistic regression analysis was used to evaluate factors affecting first-pass successful recanalization and good clinical outcomes after EVT for AIS. Variables with a *p*-value of <0.20 in the univariate analysis were included in the multivariate logistic regression analysis. The interobserver agreement for each chest X-ray feature and the presence of AoAC between the two radiologists was assessed using the Cohen kappa statistic. The degree of agreement was classified using kappa values according to the Landis and Koch recommendation as follows [25]: kappa value of 0, no agreement; 0.01–0.20, slight agreement; 0.21–0.40, fair agreement; 0.41–0.60, moderate agreement; 0.61–0.80, substantial agreement; and 0.81–1.00, almost-perfect agreement. All statistical analyses were performed using SPSS version 22 (IBM Corp., Armonk, NY, USA).

## 3. Results

A total of 193 patients (112 men and 81 women; mean age, 68.3 years; age range, 26–87 years) were analyzed. Of the 193 patients, 41 (21.2%), 119 (61.7%), and 33 (17.1%) had occlusion in the ICA, M1 and M2 segments, respectively. Stroke etiologies included atherosclerotic occlusion (*n* = 37, 19.2%), cardioembolic occlusion (*n* = 90, 46.6%), and unknown (*n* = 66, 34.2). AoAC was observed in 80 (41.5%) patients, while aortic arch types 1, 2, and 3 were observed in 46 (23.8%), 99 (51.3%), and 48 (24.9%) patients, respectively. ICAS was observed in 49 (25.4%) patients. Intravenous tissue plasminogen activator was administered to 78 (40.4%) patients. A balloon guide catheter was used in 34 (17.6%) patients. As first-line EVT strategies, stent retrievers, catheter aspiration, and a combination technique were used in 44 (22.8%), two (1.04%), and 147 (76.2%) patients, respectively. The total number of EVT attempts was one (*n* = 76), two (*n* = 43), three (*n* = 28), four (*n* = 26), five (*n* = 12), six (*n* = 3), seven (*n* = 2), eight (*n* = 2), and 12 (*n* = 1). Successful recanalization was achieved in 162 (83.9%) patients, and first-pass successful recanalization was achieved in 69 (35.8%) patients. Good clinical outcomes were achieved in 110 (57.0%) patients.

Table 1 shows a comparison of patients with AoAC and those without AoAC on chest radiographs. Patients with AoAC were significantly older (74.5 ± 7.78 vs. 63.9 ± 12.4 years, *p* < 0.001) and were more likely to have hypertension (52/80 (65.0%) vs. 43/113 (38.1%), *p* < 0.001) and atrial fibrillation (45/80 (56.3%) vs. 39/113 (34.5%), *p* = 0.003). Aortic arch type 3 was more frequently observed in patients with AoAC (34/80 (42.5%) vs. 14/113 (12.4%), *p* < 0.001). Patients with AoAC had more EVT attempts (3.04 ± 1.95 vs. 2.01 ± 1.34 times, *p* < 0.001). Procedural time was significantly longer in patients with AoAC (71.7 ± 31.2 vs. 48.7 ± 23.1 min, *p* < 0.001). Successful recanalization (60/80 (75.0%) vs. 102/113 (90.3%), *p* = 0.005) and first-pass successful recanalization (15/80 (18.8%) vs. 54/113 (47.8%), *p* < 0.001) were achieved less frequently in patients with AoAC. Postprocedural hemorrhage was observed more frequently in patients with AoAC (36/80 (45.0%) vs. 31/113 (27.7%), *p* = 0.015). Although ICH was observed more frequently in patients with AoAC (22/80 (27.5%) vs. 17/113 (15.0%), *p* = 0.045), the frequency of SAH did not differ significantly between the two groups (14/80 (17.5%) vs. 13/113 (11.5%), *p* = 0.293). Patients with AoAC had a higher incidence of good clinical outcomes (37/80 (46.3%) vs. 73/113 (64.6%); *p* = 0.013). On multivariate logistic regression analysis for factors affecting AoAC on chest radiography, including age, sex, hypertension, atrial fibrillation, occlusion site, and NIHSS at admission, age (adjusted odds ratios (OR): 1.106, adjusted 95% confidence interval (CI): 1.063–1.151; *p* < 0.001), and hypertension (2.044, 1.049–3.981, *p* = 0.036) were independently associated with AoAC on chest radiography (Table 2).

Table 3 shows a comparison of patients with first-pass successful recanalization and those without. First-pass successful recanalization was more frequently achieved for patients with M1 occlusion than for those with ICA or M2 occlusions (50/119 (42.0%) vs. 19/74 (25.7%), *p* = 0.030). Patients with first-pass successful recanalization showed a lower incidence of AoAC on chest X-ray (15/69 (21.7%) vs. 65/124 (52.4%), *p* < 0.001). Intravenous thrombolysis was administered more frequently in patients with first-pass successful recanalization (35/69 (50.7%) vs. 43/124 (34.7%), *p* = 0.033). Patients with first-pass successful recanalization had a shorter procedural time (37.0 ± 16.7 vs. 70.0 ± 27.7 min, *p* < 0.001). Postprocedural hemorrhage was more frequently observed in patients with first-pass successful recanalization (11/69 (15.9%) vs. 56/124 (45.5%), *p* < 0.001). Patients with first-pass successful recanalization had a higher incidence of both SAH and ICH (4/69 (5.8%) vs. 23/124 (18.5%), *p* = 0.016) (7/69 (10.1%) vs. 32/124 (25.8%), *p* = 0.009). Good clinical outcomes were achieved more frequently in patients with first-pass successful recanalization (53/69 (76.8%) vs. 57/124 (46.0%), *p* < 0.001). Multivariate logistic regression analysis for factors affecting first-pass successful recanalization after EVT for AIS, including sex, hypertension, occlusion site, AoAC on chest X-ray, and intravenous thrombolysis revealed that AoAC on chest X-ray (adjusted OR: 0.239, 95% CI: 0.121–0.475; *p* < 0.001) and intravenous thrombolysis (2.126, 1.126–4.014, *p* = 0.020) were independent factors (Table 4).

Table 5 compares the baseline characteristics, treatment characteristics, and radiological and clinical outcomes of patients with good clinical outcomes and those without. Patients with good clinical outcomes were significantly younger (66.2 ± 12.6 vs. 71.1 ± 10.4 years, *p* = 0.003). Hypertension was observed less frequently for patients with good clinical outcomes (47/110 (42.7%) vs. 48/83 (57.8%), *p* = 0.043). Left-side occlusion was observed less frequently for patients with good clinical outcomes (42/110 (38.2%) vs. 46/83 (55.4%), *p* = 0.020). Good clinical outcomes were less frequently achieved for patients with ICA occlusion than for those with M1 or M2 occlusions (16/110 (14.5%) vs. 25/83 (30.1%), *p* = 0.012). Patients with AoAC achieved good clinical outcomes less frequently than those who did not (37/110 (33.6%) vs. 43/83 (51.3%), *p* = 0.013). Patients with good clinical outcomes had fewer EVT attempts (2.14 ± 1.47 vs. 2.83 ± 1.89 times, *p* = 0.006), shorter procedural time (51.8 ± 24.5 vs. 66.7 ± 32.3 min, *p* < 0.001), higher rates of successful recanalization (103/110 (93.6%) vs. 59/83 (71.1%), *p* < 0.001), and first-pass successful recanalization (53/110 (48.2%) vs. 16/83 (19.3%), *p* < 0.001). Postprocedural hemorrhage (26/110 (23.9%) vs. 41/83 (49.4%), *p* < 0.001) and ICH (13/110 (11.8%) vs. 26/83 (31.3%), *p* = 0.001) were observed less frequently in patients with good clinical outcomes; however, the frequency of SAH did not differ significantly between the two groups (13/110 (11.8%) vs. 14/83 (16.9%), *p* = 0.402). Multivariate logistic regression analysis of factors affecting good clinical outcomes after EVT for AIS, including age, sex, hypertension, diabetes mellitus, atrial fibrillation, occlusion side, occlusion site, AoAC on chest X-ray, aortic arch type 3, intravenous thrombolysis, total number of EVT attempts, procedural time, successful recanalization, first-pass successful recanalization, postprocedural hemorrhage, and ICH revealed that left-side occlusion (adjusted OR: 0.381, adjusted 95% CI: 0.193–0.754, *p* = 0.006), first-pass successful recanalization (2.827, 1.329–6.018, *p* = 0.007), successful recanalization (4.088, 1.465–11.403, *p* = 0.007), and ICH (0.344, 0.150–0.791, *p* = 0.012) were independent factors (Table 6).

The interobserver agreement showed moderate agreement between the two radiologists (kappa value: 0.734 for AoAC on chest X-ray, *p* < 0.001).

## 4. Discussion

The major finding of this study was that AoAC on chest radiography was independently associated with first-pass successful recanalization after EVT for AIS. Patients with AoAC on chest radiography were also older and exhibited a higher frequency of past medical history and a type 3 aortic arch. The aortic arch type was not included in the multivariate logistic regression analysis because it is a concomitant factor rather than a factor affecting AoAC. During the procedure, these patients required more EVT attempts and longer procedural times. After the procedure, these patients showed poor recanalization outcomes and a higher risk of postprocedural hemorrhage. These findings suggest that chest radiography can provide information about vascular conditions, the difficulty of the procedure, and the radiological prognosis before EVT for AIS.

Our results showed that AoAC on chest radiography was significantly associated with a type 3 aortic arch (42.5% vs. 12.4%, *p* < 0.001) but not atherosclerotic occlusion (TOAST 1) (15.0% vs. 22.1%, *p* = 0.267). These results suggest that AoAC on chest X-ray may reflect atherosclerotic changes in a type 3 aortic arch but not intracranial atherosclerotic changes. Previous studies have reported that the pathophysiology of ICAS is different from that of extracranial atherosclerosis [24] and that ICAS was observed in patients with severe extracranial atherosclerosis [26]. This is consistent with our results, which showed AoAC was more strongly associated with aortic arch type 3 than atherosclerotic occlusion (TOAST 1).

Since EVT was recommended as the first-line treatment for AIS due to large vessel occlusion in the anterior circulation [27], recanalization rates have continued to improve with advances in devices and techniques [8]. It has been reported that EVT for AIS achieved successful recanalization in up to 90% of the cases [8,9]. However, it still fails in up to 10% of the AIS cases due to large vessel occlusion [17]. EVT failure may occur because of a tortuous vascular anatomy and ICAS-related occlusion. If the parent artery is highly tortuous, the risk of losing the stent retriever-captured thrombus increases because the stent retriever may stretch and collapse during retrieval [18]. The tortuous parent artery also reduces contact between the aspiration catheter and the thrombus, reducing the probability of thrombus aspiration [18]. If the underlying etiology of the occlusion is ICAS, EVT may damage the atheromatous surface of the occlusion site and promote platelet activation, leading to re-occlusion and EVT failure [17,19]. Therefore, vascular conditions are important for EVT in patients with AIS.

Previous studies in the fields of thoracic surgery and endovascular treatments have reported a relationship between atherosclerotic plaques in the aortic arch and perioperative stroke [23,28,29]. However, few studies have investigated the relationship between the vascular condition of the aortic arch and neurointerventional procedures. In our previous studies, AoAC on chest X-ray was significantly associated with procedural thromboembolism after coil embolization of cerebral aneurysms and procedural thromboembolism after EVT for AIS [20,21]. The current study also demonstrated the usefulness of AoAC on chest radiography as a predictor of first-pass successful recanalization after EVT for AIS. Thus, we recommend checking chest radiography before performing EVT for AIS.

The etiology of large vessel occlusion, ICAS-related large vessel occlusion, or embolism can play an important role in the response to EVT [30,31,32]. ICAS-related large vessel occlusion has a higher risk of re-occlusion, leading to multiple EVT attempts and failure [17,31,32]. Therefore, it is important to distinguish between ICAS-related large vessel occlusion and embolic occlusion to set an optimal strategy for faster and more successful recanalization [17,31]. The absence of atrial fibrillation on echocardiogram may be a surrogate marker for ICAS-related large vessel occlusion [31]. Therefore, electrocardiograms should be checked before EVT for AIS. Similarly, chest radiography is a routine examination for patients with AIS, and our study demonstrated that it played a significant role in predicting first-pass successful recanalization after EVT for AIS by showing the presence or absence of AoAC. Therefore, we believe that a chest X-ray, as well as an electrocardiogram, should be checked before EVT for AIS.

In the present study, first-pass successful recanalization was achieved more frequently for patients with M1 occlusion than for those with ICA or M2 occlusion. M1 occlusion has a relatively lower thrombotic burden than ICA occlusion; moreover, it shows a relatively straighter vessel curvature than M2 occlusion. Therefore, it is associated with a higher rate of first-pass successful recanalization than ICA or M2 occlusions [8]. Our results also showed that patients with first-pass successful recanalization had shorter procedural times, lower procedural hemorrhage rates, and higher good clinical outcome rates. This could be explained by the fact that prolonged procedural times and multiple EVT attempts may promote arterial endothelial injury and, consequently, worse neurological recovery [14].

We found a significant relationship between AoAC and first-pass successful recanalization but no significant relationship between AoAC and a good clinical outcome. This can be partially explained by the fact that a good clinical outcome was more strongly associated with successful recanalization than with first-pass successful recanalization (OR 4.088 vs. 2.827). Recent studies have reported that first-pass recanalization is strongly associated with favorable clinical outcomes [6,11,12,13,14,15,16,33,34]. Therefore, it would be meaningful to evaluate factors affecting first-pass successful recanalization.

We believe that the ability to predict first-pass successful recanalization, which indicates the procedure’s difficulty before EVT for AIS, could be helpful in deciding the strategy for EVT. Observation of AoAC on preprocedural chest radiography allows neurointerventionalists to prepare for the possibility of longer procedural times and multiple EVT attempts. If the neurointerventionalists can predict the difficulty of the procedure in advance, they can select the techniques or devices that they are familiar with rather than unfamiliar new techniques or new devices; this can help achieve better radiological and clinical outcomes.

During the initial examination of patients with AIS, brain CT angiography or MR angiography is performed, and brain CT angiography provides more information about AoAC than chest X-rays. Therefore, chest X-rays may be less useful when brain CT angiography is taken. However, neurointerventionalists try to check brain CTA focusing on intracranial lesions or carotid lesions, and they do not try to check brain CTA focusing on AoAC. Although our study was based on chest X-ray, we can suggest that we have to check AoAC on brain CT angiography as well as intracranial lesions or carotid lesions.

### Limitations

First, this was a retrospective, single-center study, and selection bias cannot be ruled out. Second, the sample size was small. Therefore, the statistical power may be low. Third, we demonstrated a significant relationship between AoAC on chest radiography and first-pass successful recanalization after EVT for AIS; however, this was not a causal relationship. Fourth, AoAC was assessed based on the presence of calcification in a qualitative rather than quantitative manner. The impact of AoAC on first-pass successful recanalization should be evaluated quantitatively or semi-quantitatively using more detailed AoAC grades. More evidence with larger sample sizes is required to confirm these preliminary results.

## 5. Conclusions

Our results suggest that AoAC on chest radiography is a useful predictor of first-pass successful recanalization after EVT for AIS; however, further evidence is required.

## Figures and Tables

**Table 1:** Comparison of patients with aortic arch calcification and those without aortic arch calcification on chest radiography before endovascular thrombectomy for acute ischemic stroke.

		Aortic Arch Calcification		*p*-Value
		Present (*n* = 80)	Absent (*n* = 113)	
Baseline characteristics	Age (years) *	74.5 ± 7.78	63.9 ± 12.4	<0.001
	Sex, male (%)	41 (51.2)	71 (62.8)	0.139
	Hypertension (%)	52 (65.0)	43 (38.1)	<0.001
	Diabetes mellitus (%)	17 (21.3)	20 (17.7)	0.580
	Atrial fibrillation (%)	45 (56.3)	39 (34.5)	0.003
	Occlusion side, left (%)	40 (50.0)	48 (42.5)	0.309
	Occlusion site			0.198
	ICA	21	20	
	M1	49	70	
	M2	10	23	
	Etiology			0.267
	Atherosclerotic	12	25	
	Non-atherosclerotic	68	88	
	Type of aortic arch			<0.001
	Type I or Type II	46	99	
	Type III	34	14	
	Intracranial atherosclerotic stenosis (%)	21 (26.3)	28 (24.8%)	0.867
	NIHSS at admission *	12.8 ± 5.79	11.4 ± 6.13	0.104
	Onset-to-door time in min *	176.1 ± 186.1	209.8 ± 222.9	0.270
	Onset-to-puncture time in min *	323.9 ± 209.3	352.8 ± 231.4	0.375
Treatment characteristics	Intravenous thrombolysis (%)	33 (41.3)	45 (39.8)	0.882
	Balloon guide catheter (%)	14 (17.5)	20 (17.7)	1.000
	Technique			0.122
	Combination	56	91	
	Non-combination	24	22	
	Total number of EVT attempts *	3.04 ± 1.95	2.01 ± 1.34	<0.001
	Procedural time in min *	71.7 ± 31.2	48.7 ± 23.1	<0.001
Radiological and clinical outcomes	Successful recanalization (%)	60 (75.0)	102 (90.3)	0.005
	First-pass successful recanalization (%)	15 (18.8)	54 (47.8)	<0.001
	Postprocedural hemorrhage (%)	36 (45.0)	31 (27.7)	0.015
	Subarachnoid hemorrhage (%)	14 (17.5)	13 (11.5)	0.293
	Intracerebral hematoma (%)	22 (27.5)	17 (15.0)	0.045
	Good clinical outcome (%)	37 (46.3)	73 (64.6)	0.013

* Data are presented as the mean ± standard deviation. ICA, internal carotid artery; NIHSS, National Institutes of Health Stroke Scale; EVT, endovascular thrombectomy.

**Table 2:** Multivariable logistic regression analysis of factors associated with the presence of AoAC on chest radiography before endovascular thrombectomy for acute ischemic stroke.

Factors	Adjusted OR	Adjusted 95% CI	*p*-Value
Age	1.106	1.063–1.151	<0.001
Hypertension	2.044	1.049–3.981	0.036

OR, odds ratio; CI, confidence interval; AoAC, aortic arch calcification.

**Table 3:** Comparison of patients with first-pass successful recanalization after endovascular thrombectomy for acute ischemic stroke and those without.

		First-Pass Successful Recanalization		*p*-Value
		Yes (*n* = 69)	No (*n* = 124)	
Baseline characteristics	Age (years) *	67.7 ± 12.7	68.6 ± 11.5	0.591
	Sex, male (%)	46 (66.7)	66 (53.2)	0.094
	Hypertension (%)	29 (42.0)	66 (53.2)	0.176
	Diabetes mellitus (%)	11 (15.9)	26 (21.0)	0.449
	Atrial fibrillation (%)	29 (42.0)	55 (44.4)	0.765
	Occlusion side, left (%)	35 (50.7)	53 (42.7)	0.296
	Occlusion site			0.032
	ICA	8	33	
	M1	50	69	
	M2	11	22	
	Etiology			0.449
	Atherosclerotic	11	26	
	Non-atherosclerotic	58	98	
	Aortic arch calcification (%)	15 (21.7)	65 (52.4)	<0.001
	Type of aortic arch			0.862
	Type I or Type II	51	94	
	Type III	18	30	
	Intracranial atherosclerotic stenosis (%)	16 (23.2)	33 (26.6%)	0.730
	NIHSS at admission *	11.8 ± 5.57	12.1 ± 6.28	0.800
	Onset-to-door time in min *	190.7 ± 192.7	198.7 ± 217.6	0.800
	Onset-to-puncture time in min *	328.5 ± 215.4	347.6 ± 226.8	0.569
Treatment characteristics	Intravenous thrombolysis (%)	35 (50.7)	43 (34.7)	0.033
	Balloon guide catheter (%)	15 (21.7)	19 (15.3)	0.324
	Technique			0.725
	Combination	54	93	
	Non-combination	15	31	
	Procedural time in min *	37.0 ± 16.7	70.0 ± 27.7	<0.001
Radiological and clinical outcomes	Postprocedural hemorrhage (%)	11 (15.9)	56 (45.5)	<0.001
	Subarachnoid hemorrhage (%)	4 (5.8)	23 (18.5)	0.016
	Intracerebral hematoma (%)	7 (10.1)	32 (25.8)	0.009
	Good clinical outcome (%)	53 (76.8)	57 (46.0)	<0.001

* Data are presented as the mean ± standard deviation. ICA, internal carotid artery; NIHSS, National Institutes of Health Stroke Scale.

**Table 4:** Multivariable logistic regression analysis of factors affecting first-pass successful recanalization after endovascular thrombectomy for acute ischemic stroke.

Factors	Adjusted OR	Adjusted 95% CI	*p*-Value
AoAC	0.239	0.121–0.475	<0.001
Intravenous thrombolysis	2.126	1.126–4.014	0.020

OR, odds ratio; CI, confidence interval; AoAC, aortic arch calcification.

**Table 5:** Comparison of patients with good clinical outcomes after endovascular thrombectomy for acute ischemic stroke and those without.

		Good Clinical Outcomes		*p*-Value
		Yes (*n* = 110)	No (*n* = 83)	
Baseline characteristics	Age (years) *	66.2 ± 12.6	71.1 ± 10.4	0.003
	Sex, male (%)	69 (62.7)	43 (51.8)	0.142
	Hypertension (%)	47 (42.7)	48 (57.8)	0.043
	Diabetes mellitus (%)	16 (14.5)	21 (25.3)	0.067
	Atrial fibrillation (%)	43 (39.1)	41 (49.4)	0.187
	Occlusion side, left (%)	42 (38.2)	46 (55.4)	0.020
	Occlusion site			0.020
	ICA	16	25	
	M1	71	48	
	M2	23	10	
	Etiology			1.000
	Atherosclerotic	21	16	
	Non-atherosclerotic	89	67	
	Aortic arch calcification (%)	37 (33.6)	43 (51.3)	0.013
	Type of aortic arch			0.179
	Type I or Type II	87	58	
	Type III	23	25	
	Intracranial atherosclerotic stenosis (%)	30 (27.3)	19 (22.9%)	0.509
	NIHSS at admission *	11.8 ± 5.57	12.1 ± 6.28	0.800
	Onset-to-door time in min *	201.5 ± 212.8	188.3 ± 203.9	0.662
	Onset-to-puncture time in min *	348.4 ± 230.7	330.7 ± 211.9	0.582
Treatment characteristics	Intravenous thrombolysis (%)	50 (45.5)	28 (33.7)	0.106
	Balloon guide catheter (%)	21 (19.1)	13 (15.7)	0.572
	Technique			1.000
	Combination	84	63	
	Non-combination	20	26	
	Total number of EVT attempts *	2.14 ± 1.47	2.83 ± 1.89	0.006
	Procedural time in min *	51.8 ± 24.5	66.7 ± 32.3	<0.001
Radiological and clinical outcomes	Successful recanalization (%)	103 (93.6)	59 (71.1)	<0.001
	First-pass successful recanalization (%)	53 (48.2)	16 (19.3)	<0.001
	Postprocedural hemorrhage (%)	26 (23.9)	41 (49.4)	<0.001
	Subarachnoid hemorrhage (%)	13 (11.8)	14 (16.9)	0.402
	Intracerebral hematoma (%)	13 (11.8)	26 (31.3)	0.001

* Data are presented as the mean ± standard deviation. ICA, internal carotid artery; NIHSS, National Institutes of Health Stroke Scale; EVT, endovascular thrombectomy.

**Table 6:** Multivariable logistic regression analysis of factors affecting good clinical outcomes after endovascular thrombectomy for acute ischemic stroke.

Factors	Adjusted OR	Adjusted 95% CI	*p*-Value
Age	0.975	0.947–1.005	0.105
Occlusion side, left	0.381	0.193–0.754	0.006
First-pass successful recanalization	2.827	1.329–6.018	0.007
Successful recanalization	4.088	1.465–11.403	0.007
Intracerebral hematoma	0.344	0.150–0.791	0.012

OR, odds ratio; CI, confidence interval.

## Data Availability

Data sharing is not applicable to this article.
